# Ecological study of socio-economic indicators and prevalence of asthma in schoolchildren in urban Brazil

**DOI:** 10.1186/1471-2458-7-205

**Published:** 2007-08-13

**Authors:** Sérgio Souza da Cunha, Mar Pujades-Rodriguez, Mauricio Lima Barreto, Bernd Genser, Laura C Rodrigues

**Affiliations:** 1 Instituto de Saúde Coletiva, Universidade Federal de Bahia, Salvador, Brazil; 2 Epicentre, medicin sans frontiers, Paris, France; 3 London School of Hygiene and Tropical Medicine, London, UK

## Abstract

**Background:**

There is evidence of higher prevalence of asthma in populations of lower socio-economic status in affluent societies, and the prevalence of asthma is also very high in some Latin American countries, where societies are characterized by a marked inequality in wealth. This study aimed to examine the relationship between estimates of asthma prevalence based on surveys conducted in children in Brazilian cities and health and socioeconomic indicators measured at the population level in the same cities.

**Methods:**

We searched the literature in the medical databases and in the annals of scientific meeting, retrieving population-based surveys of asthma that were conducted in Brazil using the methodology defined by the International Study of Asthma and Allergies in Childhood. We performed separate analyses for the age groups 6–7 years and 13–14 years. We examined the association between asthma prevalence rates and eleven health and socio-economic indicators by visual inspection and using linear regression models weighed by the inverse of the variance of each survey.

**Results:**

Six health and socioeconomic variables showed a clear pattern of association with asthma. The prevalence of asthma increased with poorer sanitation and with higher infant mortality at birth and at survey year, GINI index and external mortality. In contrast, asthma prevalence decreased with higher illiteracy rates.

**Conclusion:**

The prevalence of asthma in urban areas of Brazil, a middle income country, appears to be higher in cities with more marked poverty or inequality.

## Background

The prevalence of asthma is increasingly high in affluent countries[1]. This has been attributed by the hygiene hypothesis to lower exposure to infection resulting from improved living standards. Challenging this hypothesis, there is growing evidence that in many affluent countries the prevalence is higher among those in low socio-economic status (SES) [2-11]. These socio-economic differentials in asthma support a role of environmental factors in the development of asthma. The fact that these factors might be susceptible to change raises the possibility of identifying effective interventions to prevent asthma. In addition, if the disease burden and risk factors for asthma vary in different groups of the society, specific control measures can be targeted to these groups.

Brazil has a very unequal income distribution[12], with consequent pockets of poverty. There is some evidence that in Brazil the prevalence of asthma is higher in non-white children and in children with low maternal schooling[13,14]. In this ecological study we examined the relationship between estimates of wheezing prevalence based on surveys conducted in children in different Brazilian cities and explored whether these estimates were related to common health and socioeconomic indicators measured at the population level in the same cities.

## Methods

We searched the literature in the medical databases (PubMed and Scielo) and in the annals of scientific meetings, retrieving population-based surveys of asthma that were conducted in Brazil using the methodology defined by the International Study of Asthma and Allergies in Childhood (ISAAC). We used the following inclusion criteria: (1) surveys conducted among children aged 6–7 years or 13–14 years in Brazil between 1994 and 2003, (2) use of the official Brazilian translation of the ISAAC questionnaire [15], and (3) the study sample was chosen by probabilistic sampling and was representative of the city.

Eleven health and socioeconomic indicators were examined (Table 1). Two are measures of infant mortality rates that were calculated for each study site: "infant mortality 1" estimated at year of birth of the studied children and "infant mortality 2" at the year of the survey. All other 9 socio-economic indicators were related to the year of the survey. "Infant mortality 1" had to be calculated as these were not routine available for all cities at the time. This was done using routine data sources: the number of deaths divided by the total population in children aged <1 year per 1,000 at the year of birth of study participants (the total population was estimated as exponential extrapolation based on the census in 1980 and 1991, except for those born in 1991 [16]). For the variable "infant mortality 2" and the other 9 health and socio-economic indicators we used the mean values for the years 1991 and 2000 for the surveys conducted between 1994 and 1997, and the 2000 values for surveys conducted between 1998 and 2003. The outcome was defined as the proportion (p) of schoolchildren with reported wheezing in the last 12 months (outcome), the variance calculated as p × (1-p)/(number of participants in the survey), and the results converted to asthma prevalence (%).

**Table 1:** Definitions of health and socio-economic indicators used in the analysis

**Indicator**	**Description**
Infant mortality 1 (in the year of birth*)	Mortality per 1,000 among children <1 year in the year of birth of the study schoolchildren
Infant mortality 2 (in the survey year^2^)	Mortality per 1,000 among children aged <1 year in the year of the survey
Illiteracy rate*	Population illiteracy rate (percent) among individuals aged ≥25 years
Poverty*	Percent of the population living in poverty
Income*	Average income per capita in Brazilian currency ('reais'); unit: average for each study site
Water supply*	Percent of houses without water supply
HDI*	UN Human Development Index (HDI)
GINI*	GINI coefficient as a measure of income inequality (scale from 0 to 100)
Sanitation^†^	Percent of houses with poor sanitation (good sanitation defined as regular connection with conventional sewerage system)
Mortality for external causes^†^	Mortality for all external causes for whole population at survey year, per 10,000; number of deaths as reported for each year and population as extrapolation between censuses
Hospital beds^†^	Number of hospital beds per 10,000 inhabitants in the year survey

Notes: * Source: United Nations Development Programme, Brazil [38]; †. Source: Brazilian Ministry of Health [39]

We performed separate analyses for the age groups 6–7 years and 13–14 years. First, we conducted bivariable analysis of the relationship between asthma prevalence rates and health and socio-economic indicators by visual inspection and using the linear regression between proportion of asthma and each study variable one at a time, weighed by the inverse of the variance of each survey. For each age group, we selected the 4 variables with the lowest *P *value for multivariable analysis.

**Figure 1:**
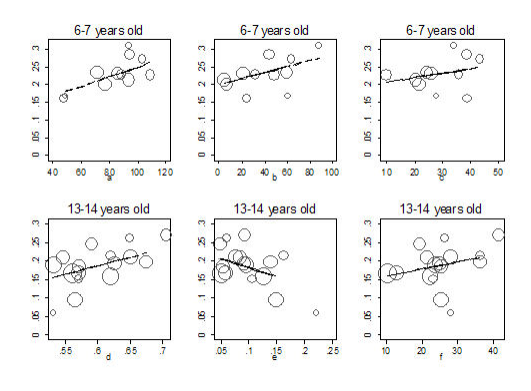
Scatter plot of the proportion of children with wheezing in the last 12 months (weighed by the inverse of the variance) against selected socioeconomic indicators by age group, (11 studies in children aged 6–7; and 16 studies in children aged 13–14 years). Y axis: proportion of children with wheezing in the last 12 months. X axis, a – mortality rate for external causes per 10,000; b – sanitation (percent of houses with poor sanitation); c – infant mortality at birth (per 1,000); d – GINI coefficient for wealth inequality; e – illiteracy rate (percent); f – infant mortality in the survey year (per 1,000). Straight line: predicted values in bivariable linear regression.

Multivariable analysis was performed using linear regression models weighed by the inverse of the variance. Fifteen linear regression models were built with all combinations of the selected four variables for each age group (all-possible-regressions selection procedure) [17]. The model with the smallest mean square error (MSE) and the highest value of adjusted R square was selected. Calculations were done with and without log transformations of the prevalence rates (outcomes); given that results were very similar (same variables were selected and similar *P *values and patterns as in the Figure 1) we only present the models without log transformation for ease of interpretation. Results from linear regression were: the regression coefficient, which can be interpreted as the prevalence points related to the change in 1 unit of the study variable; and the corresponding *P *value.

## Results

Surveys conducted in 20 cities in Brazil met the study criteria providing 13 prevalence estimates for the age group 6 to 7 year and 19 estimates for the age group 13 to 14 years [16,18-26]. When the survey results were published more than once, information from the first published report was included, as in all cases the rates were similar. We excluded reports from 5 surveys because of the following reasons: different age group (2 to 10 years) [27]; sampling procedure was not described [28]; presented only combined results for more than one study site [29]; study population was not a city but a selected area of the city and area-specific socio-economic data comparable to the other study sites were not available [30]; change of the question on wheezing in the questionnaire [31], as it has been argued that results might not be comparable with those from other surveys [32].

At visual inspection (Figure 1), variables that seemed to have a clear pattern in relation to asthma prevalence were those with statistically significant coefficients in the linear regression or that remained in the selected models. Other variables studied had an erratic pattern at visual inspection (data not shown).

In the bivariable analysis, mortality rate for external causes in the children aged 6–7 years and GINI index in those aged 13–14 years were statistically associated with wheezing (Table 2), such as that the higher the estimate the higher the prevalence of asthma found. The coefficient for mortality rate for external causes (β = 0.14; 95% C.I.: 0.01; 0.26) would mean that an increase in 1 per 10,000 mortality rate would increase asthma prevalence by 0.14 percent points in the children aged 6–7 years (or, a rise by 35.7/10,000 in mortality would increase 5 percent points asthma prevalence). The coefficient for the GINI index (scale from 1 to 100) (β = 0.46; 95% C.I.: 0.15; 0.90) would mean that a rise of 1 point in the GINI index would increase the asthma prevalence by 0.46 percent points in children aged 13–14 years (or, a rise by 10.87 points in GINI would result in an increase of 5 percent points in asthma prevalence). The relationships with asthma for 4 of the other indicators were consistent for both age groups: higher proportion of houses with bad water supply was associated with lower prevalence of asthma; while poor sanitation, infant mortality at the year of the survey, and hospital beds per 10,000 increased the prevalence of asthma. The effect of illiteracy rate, proportion of poor, average income, the human development index and infant mortality at birth of the studied children had opposite directions in the young and old age groups (Table 2).

**Table 2:** Results of the linear regression between asthma prevalence (%) and health and socio-economic indicators; outcome: percentage (%) of children with wheezing in the last 12 months in the study population

	6–7 years (11 surveys)				13–14 years (16 surveys)			
	
Health and socio-economic indicators	bivariable analysis*		multivariable analysis*		bivariable analysis*		multivariable analysis*	
	
	β	*P*	β	*P*	β	*P*	β	*P*
In the survey year								
								
Infant Mortality 1: in the survey year (per 1,000)	0.17	0.353			0.19	0.164	0.47	<0.001
Illiteracy rate (percent)	0.01	0.981			-0.49	0.096	-1.06	<0.001
Percent of poor	0.12	0.400			-0.05	0.684		
Sanitation: percent of houses with poor sanitation	0.08	0.065	0.06	0.061	0.08	0.128		
Average Income	-0.01	0.722			0.07	0.355		
Water supply	-0.16	0.350			-0.11	0.591		
HDI – Human Development Index	-32.70	0.333			21.86	0.477		
GINI coefficient for wealth inequality	10.22	0.716			0.46	0.043		
Mortality rate for external causes per 10,000	0.14	0.037	0.15	0.007	0.65	0.203		
Hospital beds per 10,000	0.58†	0.384			0.96	0.260		
								
At birth								
Infant mortality 2: at birth (per 1,000)	0.12	0.336	0.14	0.101	-0.04‡	0.512		

Notes: *bivariable linear regression with each variable one at a time and multivariable with selected variables together; † one city excluded for missing data, based on 10 surveys; ‡ one city excluded for missing data, based on 15 survey.

For children aged 6–7 years, the best multivariable model (adjusted R = 0.69) included the variables mortality for external causes rate, percent of houses with poor sanitation, and infant mortality at the year of birth. Only mortality for external causes showed a significant result (*P *= 0.007), the coefficient (β = 0.15; 95% C.I.: 0.05; 0.24) being interpreted as a rise in asthma prevalence of 0.15 percent points for each 1/10,000 increase in mortality for external causes; that is, an increase of 33.3/10,000 in mortality for external causes would increase asthma prevalence by 5 percent points.

For children aged 13–14 years the best multivariable model (adjusted R = 0.68) included two variables: illiteracy rate (β = -1.06; 95% C.I.: -1.50; -0.62) and infant mortality rate at the survey year (β = 0.47; 95% C.I.: 0.27; 0.68). An increase in the illiteracy rate of 1 percent point would decrease the prevalence of asthma by 1.06 percent points; that is, an increase in illiterate rate of 4.72 percent points would result in an decrease in asthma prevalence by 5 percent points. Conversely, An increase by 1 per 1,000 in infant mortality in the survey year would increase the prevalence of asthma by 0.47 percent points, and an increase by 10.64 in infant mortality would result in an increase by 5% percent points in asthma prevalence. No evidence of colinearity was found as standard errors of the estimators only changed slightly in different models.

## Discussion

In this ecological analysis, there is evidence that health and socioeconomic indicators were associated with asthma prevalence at ecological level, prevalence increasing with worsening of socio-economic conditions: poorer sanitation and higher infant mortality at birth (for 6–7 years) and at survey year, GINI index, external mortality; but decreased with higher illiteracy rates.

The ecological nature of the study, calls for a cautious interpretation of results, since associations with asthma found at the city level may not reflect associations at the individual level. The number of surveys is relatively small and random variation may also partly explain some of our findings. We used statistical procedures and visual inspection to guide interpretation.

Most indicators were not statistically associated with asthma prevalence. Mortality for external causes, infant mortality at the survey year (mortality 1), poor sanitation and infant mortality at the year of birth (mortality 2) remained in the best model, suggesting a higher prevalence in the cities with the worse living conditions. In contrast, higher literacy rates were associated with lower asthma prevalence. Two of the variables studied are indicators of socioeconomic inequality rather then poverty itself: mortality rate for external causes and GINI index, strongly associated in the bivariable analysis. Unless inequality in these cities is uniquely indicator of poverty at individual level, which seems unlikely, our findings suggest that asthma is also associated with population-based measures of socioeconomic disparity.

How consistent are our results with other investigations of the role of socioeconomic factors and prevalence of asthma? There is some evidence that gross national product (GNP) per capita may be associated with prevalence of wheezing in the last 12 months [33]. While high income is generally considered a positive factor for health, in more developed societies relative poverty and income seem to be more strongly related to health than absolute measures of poverty and income, specially in the field of non-communicable diseases [34]. Indeed, the evidence of association between GNP and wheezing prevalence was found to be only moderate and among children aged 13–14 years [33]. SES indicators measured at group level and not only at individual level can be associated with asthma morbidity [2,3,35,36]. SES indicators measured at an individual level are primarily related to family resources and living standard, for instances; whereas indicators at a collective level are related to, for example, community resources, social barriers and outdoor environmental exposures. Since SES indicators at individual level and at group level are likely to reflect different biological mechanisms, the use of multilevel models would be indicated to investigate the role of socioeconomic factors in the causation of asthma and their causal pathways. A further complication is that asthma is likely to be a syndrome [37], the final presentation of different aetiologies and pathways, and the relationship with SES indicators may result from the balance between effects on different types of asthma.

Finally asthma detection is very sensitive to severity; and it is possible that what we are interpreting as higher prevalence of asthma is in fact higher prevalence of severe or uncontrolled asthma. It is therefore possible that higher estimates of prevalence in the poor reflect differential access to high quality asthma care, which reduces severity. Also, the proportion of asthma cases reported by individuals of different SES is likely to differ (e.g. according to the level of education, access to health care).

## Conclusion

In conclusion, we found evidence of the association between asthma prevalence in urban centres of Brazil, a middle income country with high social disparity, with poor socio-economic indicators and inequality. To explore this better, further studies should investigate the relation between prevalence rates of different types of asthma and asthma severity, and social conditions at both the area and the individual levels. In addition, a meta-analysis based on individual and group data from the present surveys could be performed in collaboration with different research centres, as long as individual data on SES and asthma risk factors, such as schooling or income, are available.

## Competing interests

The authors declare that they have no competing interests.

## Authors' contributions

SSC and MPR originally conceived the study. SSC and BG performed the analysis. All authors have participated in the literature review, analysis plan, discussion of results, in drafting the manuscript, have read and given final approval of the version to be published.

## Pre-publication history

The pre-publication history for this paper can be accessed here:


